# Seeking an ethical and legal way of procuring transplantable organs from the dying without further attempts to redefine human death

**DOI:** 10.1186/1747-5341-2-11

**Published:** 2007-06-29

**Authors:** David Wainwright Evans

**Affiliations:** 1 Queens' College, Cambridge, CB3 9ET, UK; 2 27 Gough Way, Cambridge, CB3 9LN, UK

## Abstract

Because complex organs taken from unequivocally dead people are not suitable for transplantation, human death has been redefined so that it can be certified at some earlier stage in the dying process and thereby make viable organs available without legal problems. Redefinitions based on concepts of "brain death" have underpinned transplant practice for many years although those concepts have never found universal philosophical acceptance. Neither is there consensus about the clinical tests which have been held sufficient to diagnose the irreversible cessation of all brain function – or as much of it as is deemed relevant – while the body remains alive.

For these reasons, the certification of death for transplant purposes on "brain death" grounds is increasingly questioned and there has been pressure to return to its diagnosis on the basis of cardiac arrest and the consequent cessation of blood circulation throughout the body. While superficially a welcome return to the traditional and universally accepted understanding of human death, examination of the protocols using such criteria for the diagnosis of death prior to organ removal reveals a materially different scenario in which the circulatory arrest is not certainly final and purely nominal periods of arrest are required before surgery begins.

Recognizing the probably unresolvable conflict between allowing enough time to pass after truly final circulatory arrest for a safe diagnosis of death and its minimization for the sake of the wanted organs, Verheijde and colleagues follow others in calling for the abandonment of the "dead donor rule" and the enactment of legislation to permit the removal of organs from the dying, without pretence that they are dead before that surgery. While it may be doubted whether such a "paradigm change" in the ethics of organ procurement would be accepted by society, their call for its consideration as a fully and fairly informed basis for organ donation is to be applauded.

## Commentary

Complex organs taken from unequivocally dead people are not suitable for transplantation. This problem was thrust into prominence by the advent of cardiac transplantation in 1967. In order to avoid the legal difficulties which would have attended recognition of the fact that organs were being taken from the dying, attempts were made to redefine human death so that it could be certified prior to commencement of the organ retrieval surgery. Such redefinition required radical change in the conceptualization of the state of death as it had been known and understood in our society for hundreds of years.

The novel concept of "brain death", whose origins can be traced to experience with the maintenance of patients with irremediable brain damage in the Intensive Care Units of the 1950s and 1960s, was seized upon as a seemingly ideal basis for a definition of death for transplant purposes. In many of those patients their bodily organs remained fully functional and so in prime condition for transplantation when removed from patients in that state. Organs taken in that way, from patients certified dead on "brain death" criteria, have been the mainstay of successful transplantation programmes worldwide during the past two or three decades.

But there has never been consensus amongst philosophers about the concept; and the clinical tests, which were supposed to have the power to diagnose death of the brain while the body remains alive and functional, clearly lacked a sound scientific basis from the first. It is now widely recognized that the various forms of "brain death" are, in fact, arbitrarily defined stages in the dying process [1,2]. Consequently, the removal of vital organs from people while they are in those pre-mortal states offends fundamental ethical principles. One such principle concerns the protection of the still living, though dying, from harm by (surgical or other) assault. Such protection was assured by force of law until, in some countries, legislation was introduced to remove it for transplant purposes. Such changes in the law – to allow the certification of death on spurious grounds at some arbitary time in the dying process – do not, of course, circumvent the ethical problems. They can be addressed only by what Verheijde and colleagues [3] refer to as a "paradigm change" in the attitude of "society" towards the treatment of the dying, viz. to allow their use, with specific and completely informed consent, as sources of organs for the benefit of others.

In order to put organ donation from the "brain dead" or "brain stem dead" on a fair footing, a frank admission of what is involved in procurement procedures has been called for (among others) by Woodcock [4], by Kerridge and colleagues [5], and by Truog and Robinson [6]. They recognized that enabling legislation would be required if the "dead donor rule" were to be abandoned, i.e. if it is to be agreed and generally accepted that it is not wrong to remove organs from people before they are dead in an unambiguous sense which is not subject to conceptual and semantic manipulation. And now, as so-called "brain dead" donors are increasingly at a premium, it is encouraging to see the plea for open-ness and honesty about organ procurement procedures taken up by Verheijde and colleagues in relation to the redefinition of death on the quasi-circulatory criteria now coming into use.

Besides being a stage-managed affair, lacking in all human dignity, "donation after cardiac death" (DCD) raises major ethical concerns. Surely no one could seriously suggest that the dying are rendered de facto dead by a period of cardiac arrest as short as 2 or 5 minutes? [7]. Most of those patients could be returned to the pre-arrest state by commonplace techniques. I have personally resuscitated many patients after longer – sometimes much longer – periods of cardiac arrest. Those donors are not dead on any criteria that could be defended on scientific or other rational grounds. Nonetheless, DCD appears on the way to becoming "accepted practice" – perhaps because organ transplantation has acquired a moral imperative of its own, making it necessary and laudable to find a new way of procuring viable organs when the previously accepted basis is found wanting.

How long should one wait, after final circulatory arrest and the consequent cessation of all respiration throughout the body (and particularly the brain) before taking organs on the premise that the donor is really and truly dead (as envisaged by those donating under the "dead donor rule")? I do not know. Scientific evidence directly relevant to that question appears to be lacking at present. The more conservative will say that there should never be any urgency to diagnose and certify death. They may advocate awaiting positive signs of death [8], saying that "we do not assume death until there is not only no sign of life but every conceivable sign of death" – which means, of course, that complex organs taken thereafter will be of no use for transplantation purposes. Hence, recognizing the futility of attempts to redefine death, this call for consideration of organ procurement from the admittedly still-living, albeit assuredly dying, if this "paradigm change" can be accommodated within an acceptable system of law and ethics.
